# The Application of Wearable Glucose Sensors in Point-of-Care Testing

**DOI:** 10.3389/fbioe.2021.774210

**Published:** 2021-12-08

**Authors:** Sheng Zhang, Junyan Zeng, Chunge Wang, Luying Feng, Zening Song, Wenjie Zhao, Qianqian Wang, Chen Liu

**Affiliations:** ^1^ State Key Laboratory of Fluid Power and Mechatronic Systems, School of Mechanical Engineering, Ningbo Research Institute, Zhejiang University, Hangzhou, China; ^2^ School of Mechanical and Energy Engineering, Ningbo Tech University, Ningbo, China; ^3^ College of Chemical and Biological Engineering, Zhejiang University, Hangzhou, China

**Keywords:** glucose sensor, wearable, point-of-care testing, non-invasive, biofluids

## Abstract

Diabetes and its complications have become a worldwide concern that influences human health negatively and even leads to death. The real-time and convenient glucose detection in biofluids is urgently needed. Traditional glucose testing is detecting glucose in blood and is invasive, which cannot be continuous and results in discomfort for the users. Consequently, wearable glucose sensors toward continuous point-of-care glucose testing in biofluids have attracted great attention, and the trend of glucose testing is from invasive to non-invasive. In this review, the wearable point-of-care glucose sensors for the detection of different biofluids including blood, sweat, saliva, tears, and interstitial fluid are discussed, and the future trend of development is prospected.

## Introduction

Diabetes has become one of the most common chronic diseases caused by modern lifestyles (Bonora et al., 2021). The reduction in the number of the pancreatic β-cells leads to the shortage of insulin or the resistance from the target cells and results in type-1 diabetes or type-2 diabetes, respectively (Xiao et al., 2019a). Globally, 5–10% of the patients with diabetes have type-1 diabetes, while type-2 diabetes comprises 90–95% global diabetes (American Diabetes Association, 2014). Together with the complications, diabetes leads to a large number of premature mortalities in humans every year and is be the 7th leading cause of death according to the prediction of the World Health Organization (WHO) (Van Dieren et al., 2010; Adeel et al., 2020). As a result, continuous monitoring of the glucose level in biofluids is much needed (Kim et al., 2019; Villena Gonzales et al., 2019; Phan et al., 2021). The most traditional detected biofluid is blood. However, the collection of blood is invasive and thus causes discomfort and inconvenience for the users. Furthermore, invasive collection hinders continuous monitoring of blood glucose (Lee et al., 2018). Consequently, more research studies are toward sweat (Bariya et al., 2018), saliva (Mani et al., 2021), tears (Guo et al., 2021), and interstitial fluid (Kim et al., 2018) as alternatives to develop non-invasive, continuous, wearable, and point-of-care monitoring of glucose (Yoon et al., 2020).

Unlike the traditional diagnostic tests which need to analyze the sample in a laboratory and obtain the results after hours and even several days, point-of-care testing (POCT) has been applied in resource-limited areas and hospital emergency rooms (Narinx et al., 2020; Raiten et al., 2020; Holmström et al., 2021). Although the pain brought by blood collection can be alleviated, it is not suitable for continuous blood glucose monitoring, especially during exercise (Muñoz Fabra et al., 2021). Besides the fast analysis time and less pain for patients, compared with the routine diagnostic test, point-of-care testing is normally easy to use, portable, and inexpensive and has less risk for infections (Darwish et al., 2018; Nichols, 2020; Shrivastava et al., 2020). Therefore, point-of-care testing displays great potential not only in continuous, long-term monitoring of various kinds of diseases including diabetes (Zhang et al., 2020) but also in food safety analysis and environmental monitoring (Xu et al., 2020; Zaczek-Moczydlowska et al., 2021).

In this review, wearable glucose sensors in point-of-care testing are divided into six classifications according to the sensing target: blood, sweat, saliva, tears, interstitial fluid, and urine. Additionally, the prospect of wearable glucose sensors toward POCT is outlook.

## Biofluids Detected

### Blood

The glucose level in blood is the most traditional indicator and the gold standard for diabetes (Lee et al., 2018). Although blood testing is invasive, blood glucose testing possesses satisfactory sensitivity both for testing in the laboratory and the finger prick test at home, is reliable and low cost, and is a well-established technique (Wang and Lee, 2015). Therefore, blood glucose is regarded as the gold standard for diabetes diagnosis, and the wearable sensors toward the detection of blood glucose play a significant part in the health care of diabetes patients (Makaram et al., 2014). Blood glucose testing is mostly used in our daily life and has also been applied for point-of-care testing. However, the sensitivity of commercial blood glucose instruments is not high enough so the patients should test their blood glucose level several times to make the result precise. The commercial blood glucose instruments are unwearable and not portable, thus causing inconvenience for users. Some studies have been conducted to address these concerns.

**FIGURE 1:**
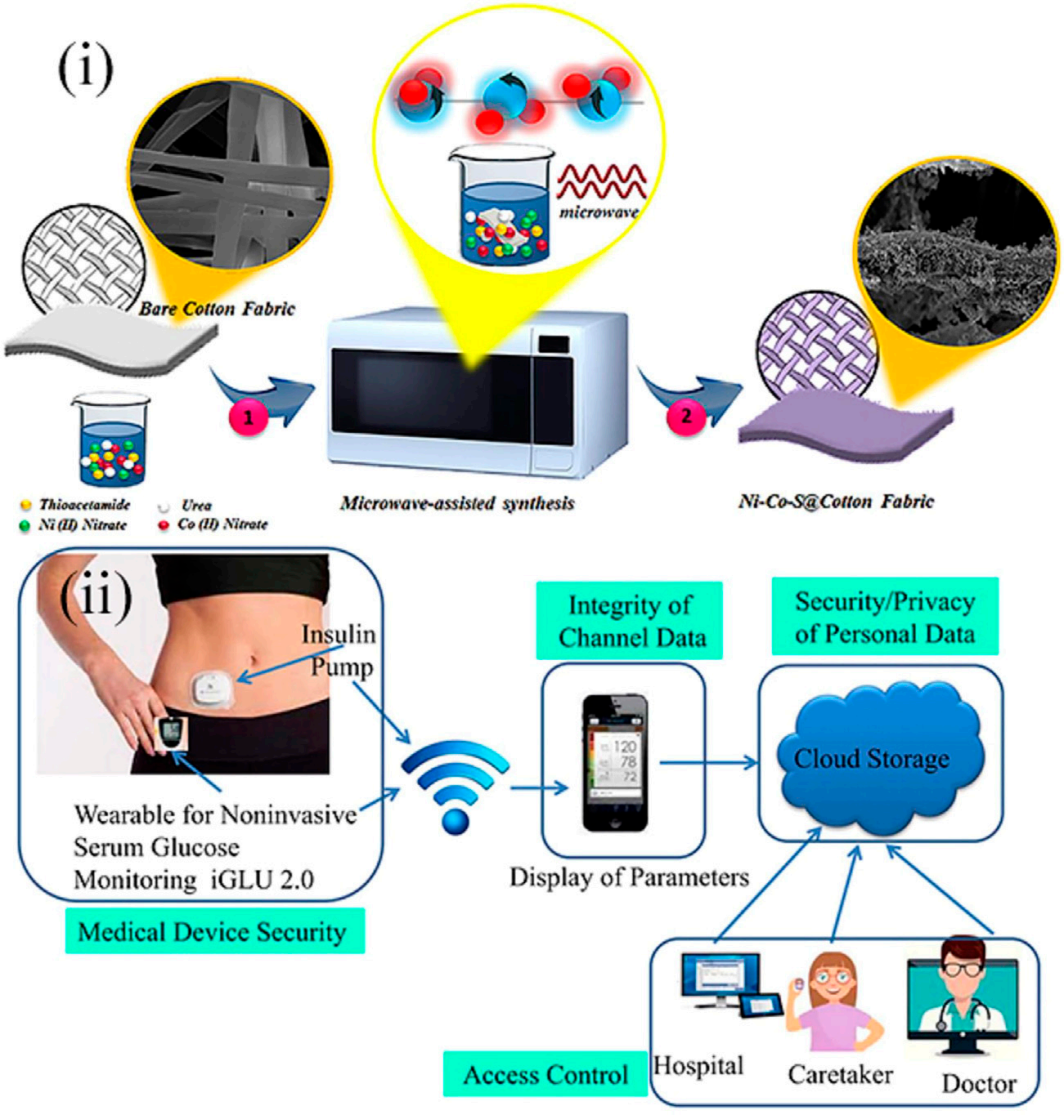
**(i)** Schematic diagram showing the fabrication process of Ni-Co-S@CF electrodes (Hekmat et al., 2021). **(ii)** Prospective toward the long-term glucose monitoring application of point-of-care wearable glucose sensors, iGLU 2.0 (Joshi et al., 2020).

For example, in Hekmat et al., a point-of-care platform toward the sensing of blood glucose was constructed (Hekmat et al., 2021). Using a micro-assisted method, ternary nickel cobalt sulfide was decorated on the commercial cotton fabrics to form the Ni-Co-S@CFs electrodes (Figure 1(i)). The method was facile and just needs one step. The unique structure of the electrode enabled the sensor with satisfactory repeatability, long-term stability, outstanding selectivity, low detection limit, and a wide sensing range, and it can be used in alkaline media. Besides, this sensor could also detect the glucose level in saliva. Although all these advantages and many other evolutions have been made for the blood glucose sensor, the traditional blood sample collection method is invasive and thus causes discomfort for the patients and increases the risk of being infected (Lee et al., 2018). The invasive collection method also prevents the point-of-care detection from being continuous (Rodin et al., 2019). These are also shortcomings in the commercial blood glucose instruments. As a result, Joshi et al. designed a new wearable point-of-care device for the non-invasive and continuous measurement of blood glucose (Joshi et al., 2020). The wearable sensor was based on near-infrared (NIR) spectroscopy and incorporated with an Internet of Medical Things (IoMT) to sense, transmit, and restore the data from patients on the cloud. In this way, the data could be available for patients and medical personnel, and continuous monitoring of glucose could be achieved [Figure 1(ii)]. The following experiments demonstrated that the point-of-care device was cost-efficient and precise and could detect blood glucose in a wide range from 80 to 420 mg/dl. The device was called iGLU 2.0 and indicated a broad prospect in smart health care in the future.

Apart from blood, sweat (Bariya et al., 2018), saliva (Mani et al., 2021), tears (Guo et al., 2021), interstitial fluid (Kim et al., 2018),and urine (Zhang et al., 2021a) can also be indicators for diabetes as their chemistry is closely related to blood and thus all being the target for the point-of-care testing. Moreover, compared with blood, the collection of these body fluids does not need to destroy the stratum corneum so that is easier to achieve non-invasive and continuous detection of glucose. As a result, more researchers tend to fabricate non-invasive point-of-care wearable glucose sensors toward the detection in these body fluids, especially sweat.

### Sweat

The research studies toward the point-of-care wearable sweat glucose sensor are the most common among the other body fluids (Morse et al., 2016; Lee et al., 2017; Thulasi et al., 2017; Bhide et al., 2018a; Bhide et al., 2018b; Xuan et al., 2018; Zhang et al., 2018a; He et al., 2019; Xiao et al., 2019b; Veeralingam et al., 2020; Bauer et al., 2021; Zheng et al., 2021) because compared with saliva, tears, and interstitial fluid, sweat is easier to access and will not cause discomfort for patients and the detection of sweat exhibits less risk for infection (Arakawa et al., 2016; Yu et al., 2019; Zheng et al., 2021). Despite all these advantages, there exist some disadvantages/challenges to the application of point-of-care wearable sweat glucose sensors. Without iontophoretic stimulation, sampling will be irregular for individuals during the day (Heikenfeld, 2016) and the sample production rate will be extremely low (Sonner et al., 2015). The method to dissolve these problems is to increase the sensitivity of the sweat glucose sensor so that the volume of sweat samples needed for glucose detection can be decreased. In order to increase the sensitivity, researchers try to use filter papers and distinct classification of films, patches, and nanosheets as substrates of the sweat glucose sensors. Furthermore, the thickness of these basic materials is extremely low, especially for the nanosheets, a kind of two-dimensional material, so that the size of the sweat glucose sensors decreases, and thus easier to achieve wearability.

**FIGURE 2:**
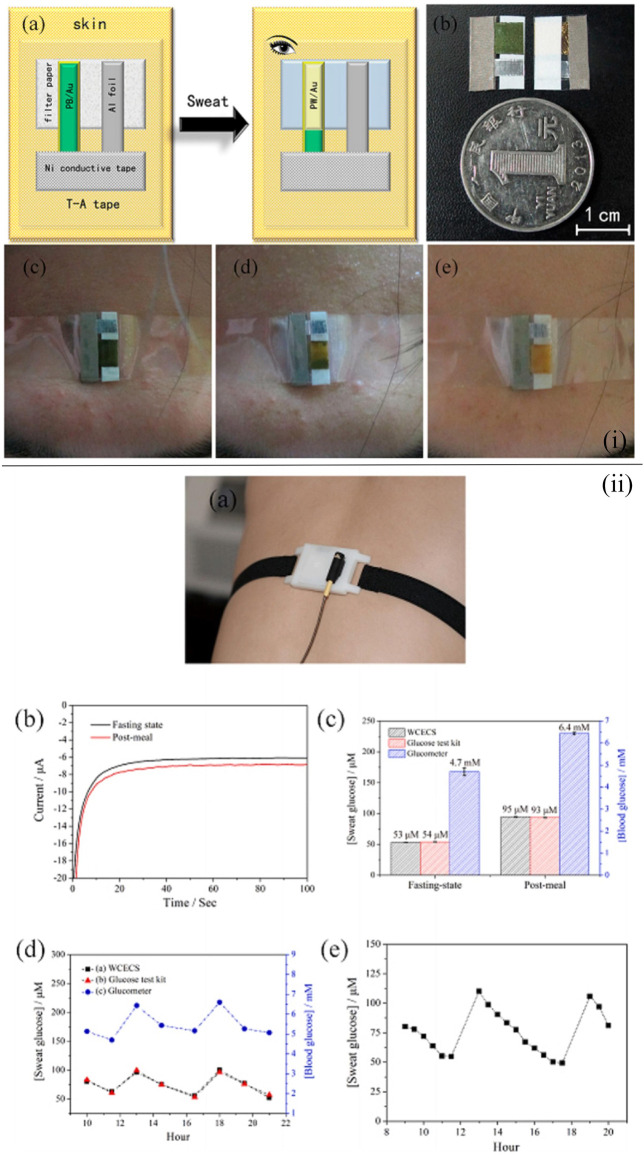
**(i)** Schematic illustration demonstrating the fabrication of the self-powered sensor for point-of-care sweat monitoring **(A)**, a photograph of the fabricated sensor **(B)**, and photographs of the point-of-care sensor on the forehead of the volunteer when exercising 0 min **(C)**, after 29-min exercise **(D)**, and after 32-min exercise **(E)** (Zhang et al., 2018a). **(ii)** Working of the WCECS in real time on the body. **(A)** Photograph of the WCECS attached on the back of a human subject. **(B)** EC response of sweat glucose in the post-meal and fasting state. **(C)** Contrast of the sweat glucose concentrations sensed by the WCECS glucometer and glucose test kit. **(D)** Comparison of the glucose concentrations detected in 1 day by the glucometer, glucose test kit, and WCECS. **(E)** Evaluation of durability of the WCECS (Zheng et al., 2021).

Paper-based substrates are one of the optimal basis materials for the wearable glucose sensor, and there exist a large number of wearable point-of-care glucose sensors based on the filter paper fabricated by researchers (Cho et al., 2017; Zhang et al., 2018a; Zhang et al., 2019; Zheng et al., 2021). For instance, a self-powered, low-cost, and facile wearable sensor for the point-of-care detection of glucose levels in sweat was reported to be developed by Zhang et al. (2018a). Au/multiwalled carbon nanotube (MWCNT) glucose dehydrogenase was applied to monitor the glucose in sweat [Figure 2(i)]. The use of Au/Prussian blue indicating electrodes enabled the users to regard the color change as the indicator of glucose level. As a result, there was no need for other instruments, thereby reducing the weight and cost of the sensor. The electrodes were deposited on the filter paper to improve the sensing performance of the sensor. The sensing component was assembled with an energy component by a transparent adhesive tape so that the sensor could be self-powered and display remarkable sensing performance, holding promise in the application of point-of-care testing. Similarly, Zheng et al. fabricated a point-of-care device based on filter paper and carbon nanotubes (CNTs) for the detection of the glucose level in sweat (Zheng et al., 2021). A new wearable cloth-based electrochemical sensor (WCECS) containing superior sweat collection and transport channel was applied to analyze the glucose level in sweat. Sweat was transported into a cloth-based chip which was constructed by the facile and low-cost screen printing technology [Figure 2(ii)]. Therefore, the sensor not only exhibited prominent stability, reproducibility, and selectivity but also was low cost and can monitor for 9 h continuously. The paper-based point-of-care device (PAD) with the cotton thread as the microchannel for sweat harvest is a satisfactory choice to sense the glucose level in sweat. In Xiao et al., a microfluidic thread/paper-based analytical device (μTPAD) made of filter paper and a cotton thread was fabricated (Xiao et al., 2019b). By optimizing the amounts of reagents and enzymes on the functionalized filter paper, the highest colorimetric sensing performance toward sweat glucose was found, while the wicking properties of the cotton thread were also optimized with the assistance of the oxygen plasma. Additionally, by integrating with an arm guard and the application of a smartphone, a low-cost, non-invasive, and easy-to-use point-of-care glucose sensing system with excellent compatibility and wearability was established.

**FIGURE 3:**
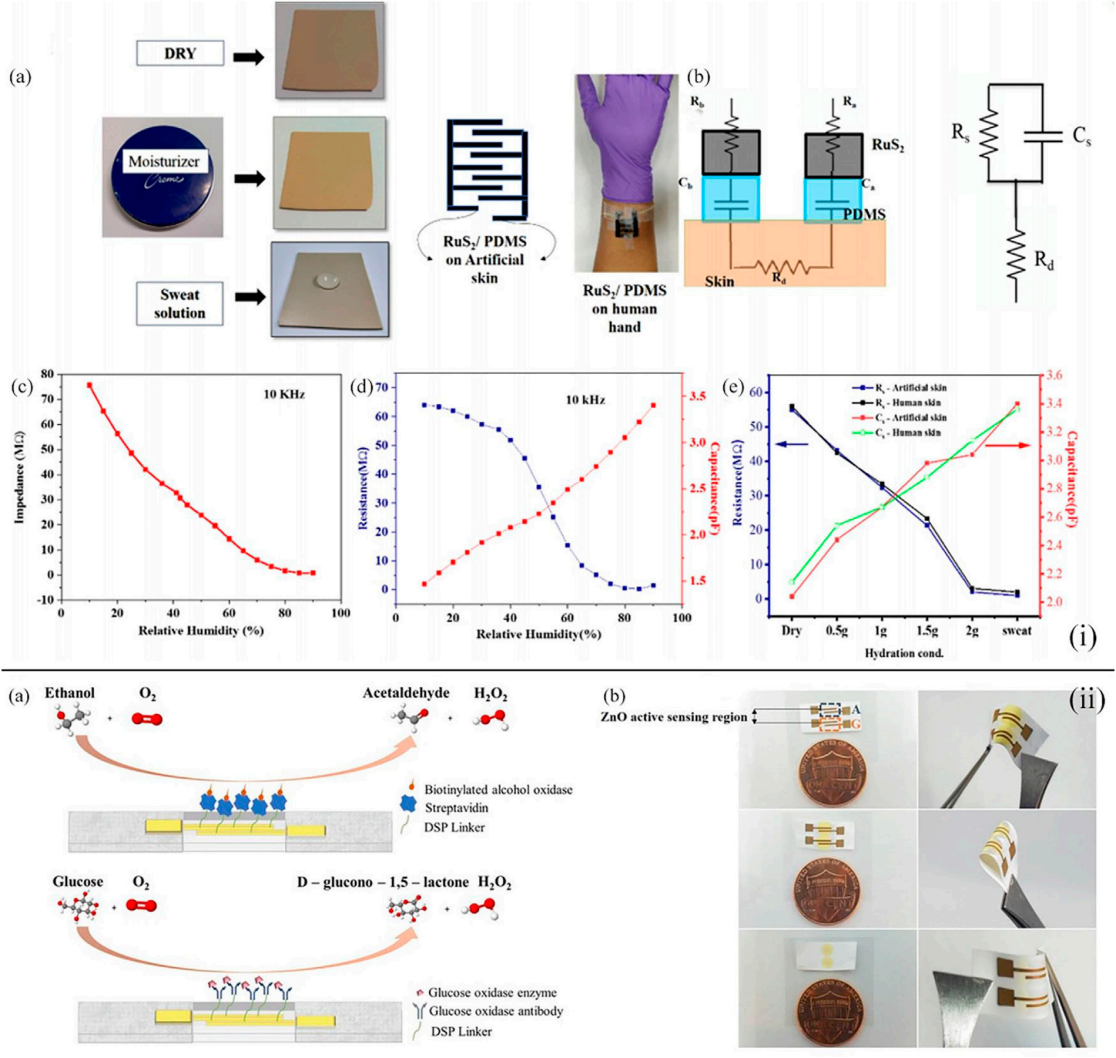
**(i) (A)** Different skin conditions for artificial skin and the sensor tied on the human skin. **(B)** Equivalent circuit representation of the designed RuS_2_/PDMS-based hydration sensor. **(C)** Impedance value detected at the increase in humidity conditions on artificial skin at an alternating current frequency of 10 kHz. **(D)** Capacitance and resistance as the function of the increase in relative humidity conditions when the sensor was tied on the human skin. **(E)** Capacitance and resistance values of the human skin and artificial skin at distinct hydration environments (Veeralingam et al., 2020). **(ii) (A)** Immunoassay with the ability of the combined monitoring of glucose and alcohol. **(B)** Sweat sensor array displaying fluid confinement in the active detection region, size comparison with one cent, and the flexibility of sensor (Bhide et al., 2018a).

Besides the filter paper, distinct kinds of films can also be the basic materials of the wearable point-of-care device toward the sensing of sweat glucose (Bhide et al., 2018a; Veeralingam et al., 2020; Müsse et al., 2021). For instance, Veeralingam et al. first reported a wearable multifunctional sensor platform enabled with artificial intelligence/machine learning (AI/ML) (Veeralingam et al., 2020). This sensor could continuously monitor pH and glucose levels in sweat and the hydration level of the skin with high speed and accuracy. A facile hydrothermal method was applied to synthesize RuS_2_ nanoparticles (NPs), and the RuS_2_ NPs were deposited on the PDMS film substrates by layer-by-layer spin coating technology. The application of K-nearest neighbors (KNN) which is based on artificial intelligence in the open-source microcontroller board (QueSSence) greatly ensured the precision and fast data acquisition of glucose, and it was demonstrated that the wearable sensor platform possessed prominent reusability and stability at room temperature [Figure 3(i)]. Moreover, Bhide et al. integrated zinc oxide films into a flexible nanoporous electrode to form an electrode system (Bhide et al., 2018a). The sensing mechanism of the sensor was to measure the impedance change resulting from the glucose bonding on the surface of the electrode, which was detected by electrochemical impedance spectroscopy [Figure 3(ii)]. Glucose oxidase enzyme and alcohol oxidase enzyme were applied to functionalize the surface of the zinc oxide film electrodes to improve the sensing range of the wearable sweat glucose sensor from hypo- to hyperglycemia (50–100 mg/dl), and when compared with the data of a commercial breathalyzer, the calibration of the sensor was excellent. As a result, this lancet-free glucose sensor could monitor glucose levels with a low volume of sweat and show great accuracy, wide sensing range, and low detection limit in point-of-care testing.

**FIGURE 4:**
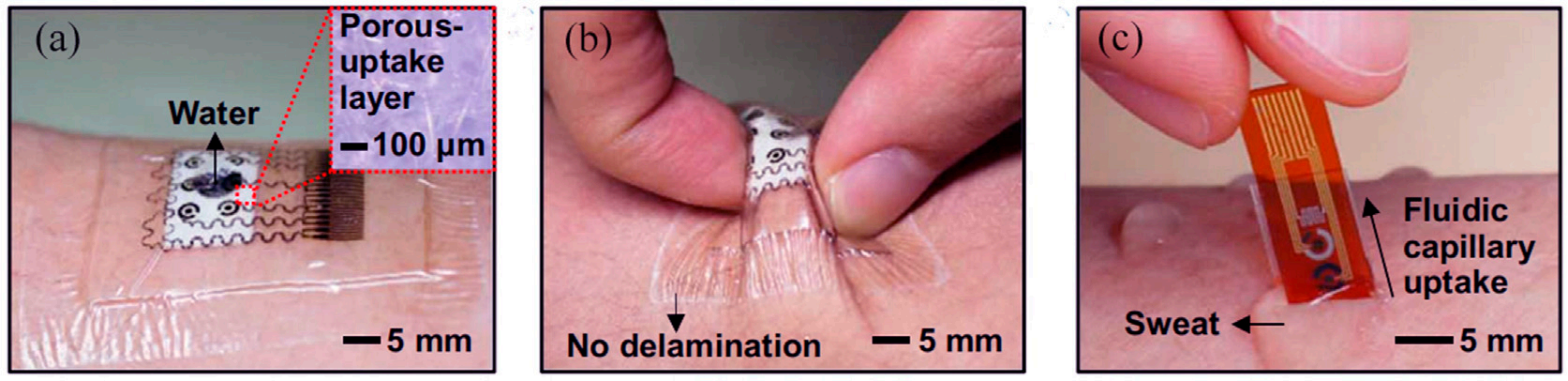
**(A)** Photograph of the wearable patch-based glucose sensor with a waterproof band and a sweat collection layer. **(B)** Photograph of wearable patch-based glucose sensor under deformation. **(C)** Optical image of disposable patch-based glucose sensor on the human skin with sweat (Lee et al., 2017).

A patch-based point-of-care device for the monitoring of glucose levels in sweat was reported to be proposed by Lee et al. (2017). The unique multilayer patche structure minimized the sensor and remarkably increased the sensing efficiency. Besides, the porous structure provided a large number of electrochemical sites and thus higher enzyme immobilization (Figure 4). According to the glucose level detected by the glucose sensors, the device could also release the precise, controlled, and multistage drug for the patients. Hyaluronic acid hydrogel microneedles were coated with phase change materials and two distinct temperature-responsive phase change nanoparticles to achieve feedback transdermal therapy. This wearable point-of-care device not only provides a novel structure for the monitoring of sweat glucose with high efficiency but also paves a way for the closed-loop solution of diabetes management.

**FIGURE 5:**
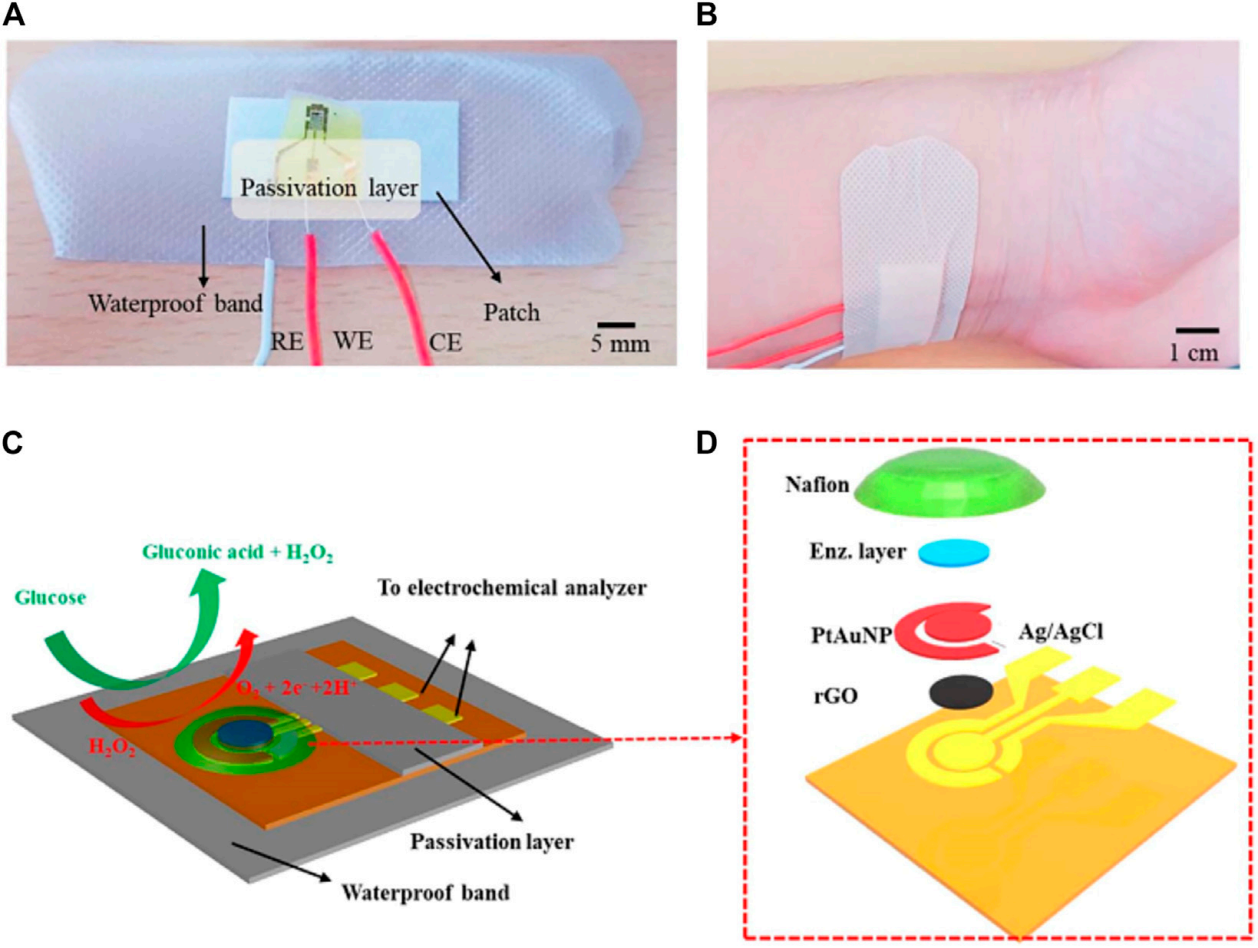
Optical images and schematic diagrams displaying the wearable point-of-care biosensor toward the detection of glucose in perspiration. Photographs **(A,B)** of the constructed wearable sensor. Schematic illustration of the whole wearable point-of-care sensor **(C)** and exploded view **(D)** (Xuan et al., 2018).

Nanosheets, two-dimensional (2D) materials, display prominent catalyst properties due to their high surface-to-volume ratio and thus numerous electrocatalyst sites (Zhang et al., 2017; Liu et al., 2021a; Zhang et al., 2021b). Consequently, innovative research studies toward the application of 2D nanomaterials are increasing, especially in the field of sensing application, including the wearable point-of-care glucose sensor for the detection of sweat (Zhang et al., 2018b; Xuan et al., 2018; Guo et al., 2019; Yang et al., 2019). In Xuan et al., reduced graphene oxide (rGO) nanosheets were coated with platinum and gold nanoparticles to form rGO nanocomposites as the working electrode (Xuan et al., 2018). After being microfabricated, the nanostructures were micropatterned on a flexible polyimide substrate by a low-cost and facile procedure. The working electrode was also integrated with chitosan glucose oxidase composites to achieve sensing of glucose. The unique structure and processing method endowed the point-of-care device with a large detection range, remarkable amperometric response to glucose, fast response, high linearity, and high sensitivity (Figure 5).

Although plenty of research studies have been made for sweat glucose sensing, several challenges prevent wearable point-of-care sweat glucose sensors from being applied in daily life besides the sampling problems. The skin can act as a contamination source, leading to the contamination of sweat samples, and new sweat can be mixed and contaminated by the old sweat (Liu et al., 2020). Moreover, a huge change in sweat pH (between 4.5 and 7.0) and the active analyte channels that exist in eccrine glands will make a skew of glucose concentration in sweat (Heikenfeld, 2016).

### Saliva

Saliva is a very attractive biofluid toward point-of-care non-invasive monitoring applications as researchers found that saliva collected from diabetics has higher glucose concentration values (Liu et al., 2015; Zhang et al., 2015). Additionally, the simple non-invasive process of saliva collection and the needlessness of sample pretreatment make it possible for saliva’s extensive application in wearable point-of-care sensors (Arakawa et al., 2016). However, in several cases, saliva needs to be treated by either filtration or dilution (Ji and Choi, 2015). Besides convenience, saliva is a challenging biofluid for electrochemical measurements. Saliva is a kind of ultrafiltrate of blood and contains mostly water (Czumbel et al., 2020). As a result, the concentration of biomarkers is always much low in saliva, which is the most significant shortcoming of saliva as a detection biofluid (Chiappin et al., 2007; Miočević et al., 2017). Moreover, the specific confounds by the oral cavity also influences saliva as the point-of-care monitoring biofluid (Miočević et al., 2017). Researchers are trying their best to dissolve these problems.

**FIGURE 6:**
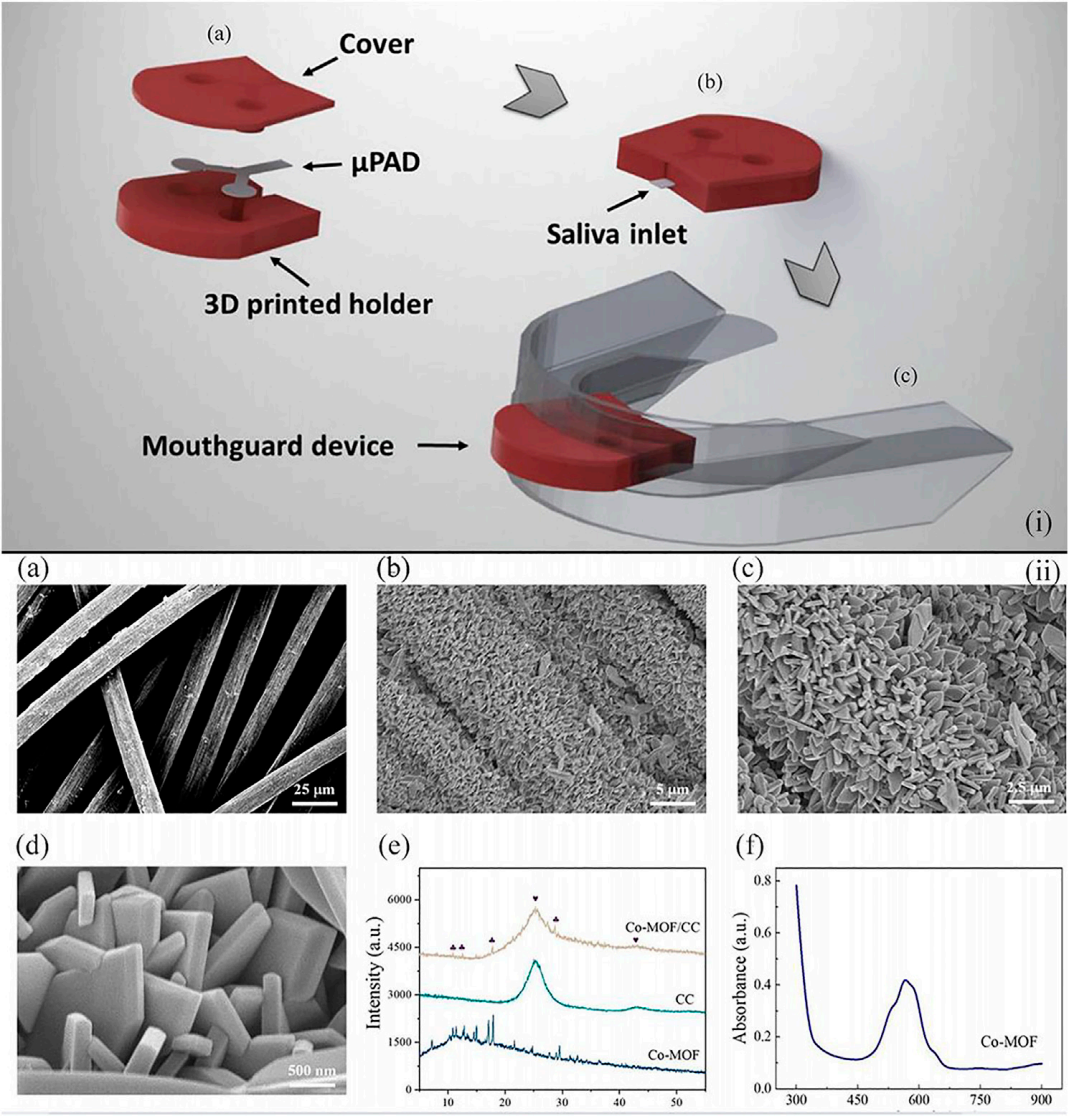
**(i)** Schematic illustration indicating the μPAD assembled into a mouth guard by a 3D-printed holder to form the wearable paper-based devices for point-of-care testing of glucose concentration in saliva. **(A)**, **(B)**, and **(C)** illustrate the arrangement of the μPAD in the 3D-printed holder, the final device before and after integration into the mouth guard, respectively (de Castro et al., 2019). **(ii)** Photographs **(A)** of the button-sensor, and schematic illustration showing **(B)** the assay procedure (Wei et al., 2021).

For example, Castro et al. developed a microfluidic paper-based wearable sensor for glucose monitoring (de Castro et al., 2019). The reported device integrated microfluidic paper-based devices (μPADs), the 3D printed holder, and the silicone mouthguard for the realization of salivary diagnostics. A mixture of 4-aminoantipyrine (AAP) and 3,5-dichloro-2-hydroxybenzenesulfonic acid (DHBS) as a chromogenic solution was used in μPADs, and μPADs were fabricated through simple and low-cost technologies. The 3D-printed holder made insulation between the mouth and the reagents, which eliminated the risk of the water-soluble chemical assay reagents in these wearable sensors to the health of patients. Without any pretreatment process, this low-cost and partially recyclable wearable sensor represented a major step forward in the field of point-of-care testing devices [Figure 6(i)].

Apart from colorimetric measurements (Tian et al., 2016; de Castro et al., 2019), the non-enzymatic electrocatalytic reaction based on the metal–organic framework (MOF) is another stable way for glucose sensing, which displays higher sensitivity (Ling et al., 2020; Wang et al., 2020). In particular, Wei group has made significant progress toward non-enzymatic quantitative detection of glucose (Wei et al., 2021). The team developed a cobalt metal–organic framework–modified carbon cloth/paper (Co-MOF/CC/paper) hybrid button-sensor as the simple and portable electrochemical analytical chip. Co-MOF was an artificial nanozyme featuring low cost, easy production, and high environment tolerance and was an ideal succedaneum of the commonly used enzyme in glucose detection. In addition, the flexible Co-MOF/CC sensing interface of this reported sensor, which was effectively integrated with the patterned paper, provided adequate catalytic sites and a high specific area [Figure 6(ii)]. Compared to the glucose detected in serum, this portable button-sensor shows a comparable accuracy to that of a commercial glucometer and presents a promising platform for wearable POCTs.

### Tears

Recently, the glucose level in tears has attracted great attention in wearable point-of-care glucose sensors. It is confirmed that tears participate in the metabolism of glucose in the human body, and the glucose concentration in tears is a positive correlation with the glucose level in blood (Chen et al., 1996; Chatterjee et al., 2003). Besides, myopia nowadays has become a global health issue and the prevalence is remarkably high, especially in east Asia (Morgan et al., 2012). Wearing contact lenses is one of the most favorite ways to correct vision. As a result, the smart contact lens with the ability to collect tears and then monitor the glucose in tears has become a welcome wearable point-of-care device for glucose detection (Yao et al., 2011; Elsherif et al., 2018; Lin et al., 2018; Park et al., 2018).

**FIGURE 7:**
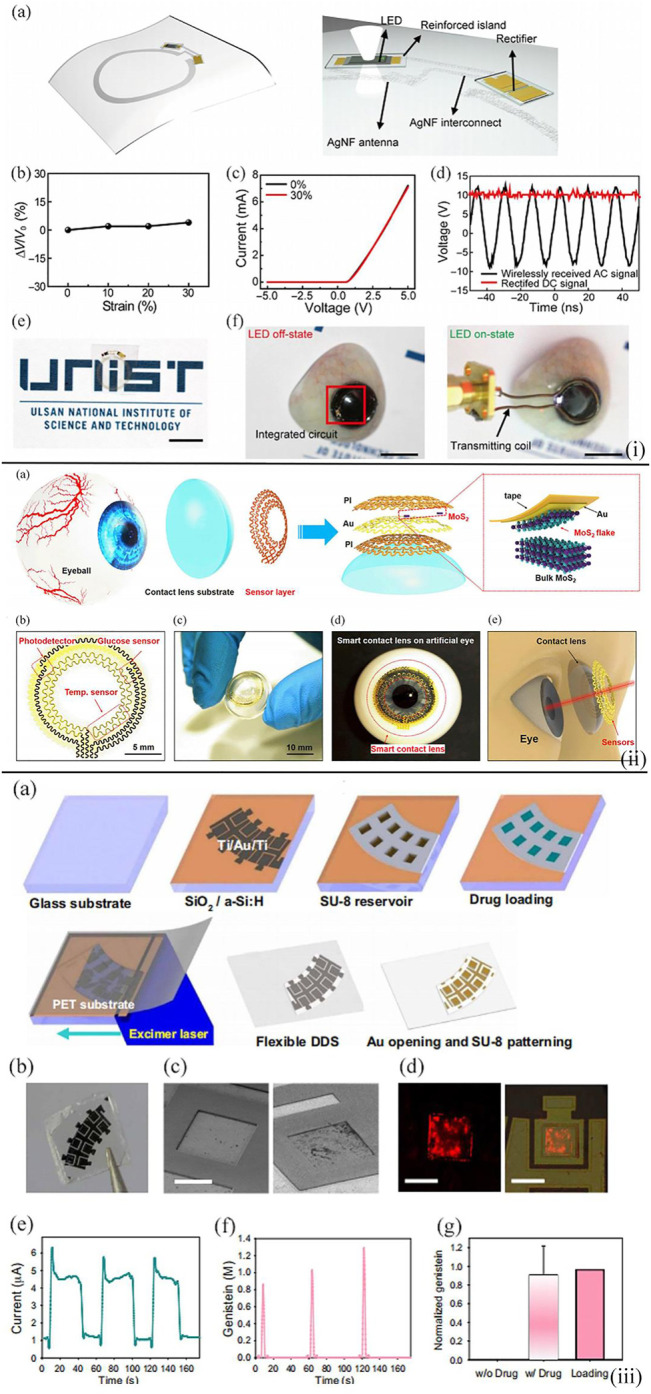
**(i)** Wireless representation circuit on the substrate. **(A)** Schematic diagram illustrating the wireless display circuit. The stretchable, transparent AgNF-based antenna and interconnects are in an elastic area, while the LED and rectifier are located in the reinforced area. **(B)** Relative change in transmitted voltage by antenna versus the applied strain. **(C)** Characterizations of Si diode on the hybrid substrate by using 0 and 30% in tensile strain. **(D)** Rectified properties of the constructed rectifier. **(E)** Optical image of wireless display circuit on the hybrid substrate. Scale bar, 1 cm. **(F)** Photos **(left, off-state; right, on-state)** of operating wireless display with lens shape located on the artificial eye. Scale bars, 1 cm (Ruan et al., 2017). **(ii)** Design of the structure of a smart contact lens with ultrathin MoS_2_ transistor–based serpentine mesh sensor system. **(A)** Schematic diagram showing the distinct layers of smart contact lens structure placed onto an eyeball. The dashed region highlights the method of gold-mediated mechanical exfoliation for the fabrication of monolayer MoS_2_. **(B)** Images of the sensor structure and serpentine electrode. **(C)** Photograph of a dome-shaped PDMS substrate with the sensor layer on it. **(D)** Photograph of an artificial eye with the sensing system attached to it. **(E)** Schematic diagram illustrating the smart contact lens and the sensors placed on the eyeball (Guo et al., 2021). **(iii)** On-demand drug delivery applying an f-DDS. **(A)** Schematic diagram displaying the construction process of f-DDS. **(B)** Photographic image of f-DDS. **(C)** SEM images of f-DDS before and after the gold electrochemistry experiment. Scale bar, 250 μm. **(D)** Confocal fluorescence microscopic images of rhodamine B dye released from drug reservoirs. Scale bars, 300 μm **(left)** and 500 μm **(right)**. **(E)** Change of current for the f-DDS. **(F)** Released levels of genistein in a pulsatile manner. **(G)** Normalized content of genistein released from the reservoirs (*n* = 6) in comparison with the initial loading content (Keum et al., 2020).

Ruan et al. reported the fabrication of an attached lens based on a gelated colloidal crystal for point-of-care tear glucose detection (Ruan et al., 2017). The novel glucose sensor was made by embedding a crystalline colloidal array in a matrix of hydrogel and amounted on the rigid gas permeable lens [Figure 7(i)]. With the change in the glucose level in tears from 0 to 50 mM, the sensing contact lens could diffract visible light with distinct wavelengths from 567 to 468 nm accordingly and thus showed different colors from reddish yellow to blue. This novel point-of-care sensor exhibited a low detection limit of 0.05 mM, and with the assistance of the contact lens, the device also showed superior portability and biocompatibility.

In Guo et al., a multifunctional smart contact lens based on MoS_2_ transistors were developed (Guo et al., 2021). On the PDMS lens substrate, there was a glucose sensor based on MoS_2_ nanosheets for the direct detection of the glucose concentration in tear, a photodetector to receive optical information, and a temperature sensor based on Au to monitor the potential corneal disease. This serpentine mesh structure enabled the sensor to contact with tears and was mounted on the contact lens directly so that the sensing sensitivity would be increased and blinking or vision would not be interfered [Figure 7(ii)]. Moreover, the following tests demonstrated the remarkable biocompatibility of the lens, and thus, this smart contact lens showed great potential as the next-generation point-of-care wearable soft device for personal health care.

The recent research direction toward the point-of-care tear glucose sensors is not only to diagnose diabetes and related complications but also to assist with therapy. In Keum et al., a smart lens device was attached to a polymer with excellent biocompatibility (Keum et al., 2020) [Figure 7(iii)]. This point-of-care device consisted of ultrathin soft circuits and a microcontroller for the detection of glucose concentration in tears, drug delivery, data transmission, and wireless power supply. It was demonstrated that the concentration of tear glucose detected by the contact lens was validated by blood glucose, and drugs could be triggered to deliver for the diabetic retinopathy therapy. This work first constructed a contact lens with the capability of biometric analysis in combination with drug delivery and paved the way for personal health-care and medical devices with a combination of diagnosis and therapy at the same time in perspective view.

The most significant challenge for the tear glucose sensors is the power supply. As the human eye is delicate, the power supply device must be soft, and the external power supply applied in most research studies nowadays will bring great discomfort for users (Bandodkar and Wang, 2014). Although ascorbate (Falk et al., 2013) and lacrimal glucose (Falk et al., 2012) have been demonstrated as usable energy supplies in biofuel cells, further studies need to be performed for future applications.

### Interstitial Fluid

Interstitial fluid is found between the cells of the body that provides much of the liquid environment of the body. Since the interstitial fluid (ISF) contains a higher glucose concentration value, through related technologies, a non-invasive blood glucose sensor based on the interstitial fluid (ISF) can obtain higher sensitivity and accuracy (Potts et al., 2002; Bandodkar et al., 2015; Lee et al., 2018; Lipani et al., 2018). Therefore, it is also a very attractive biofluid toward point-of-care non-invasive monitoring applications.

**FIGURE 8:**
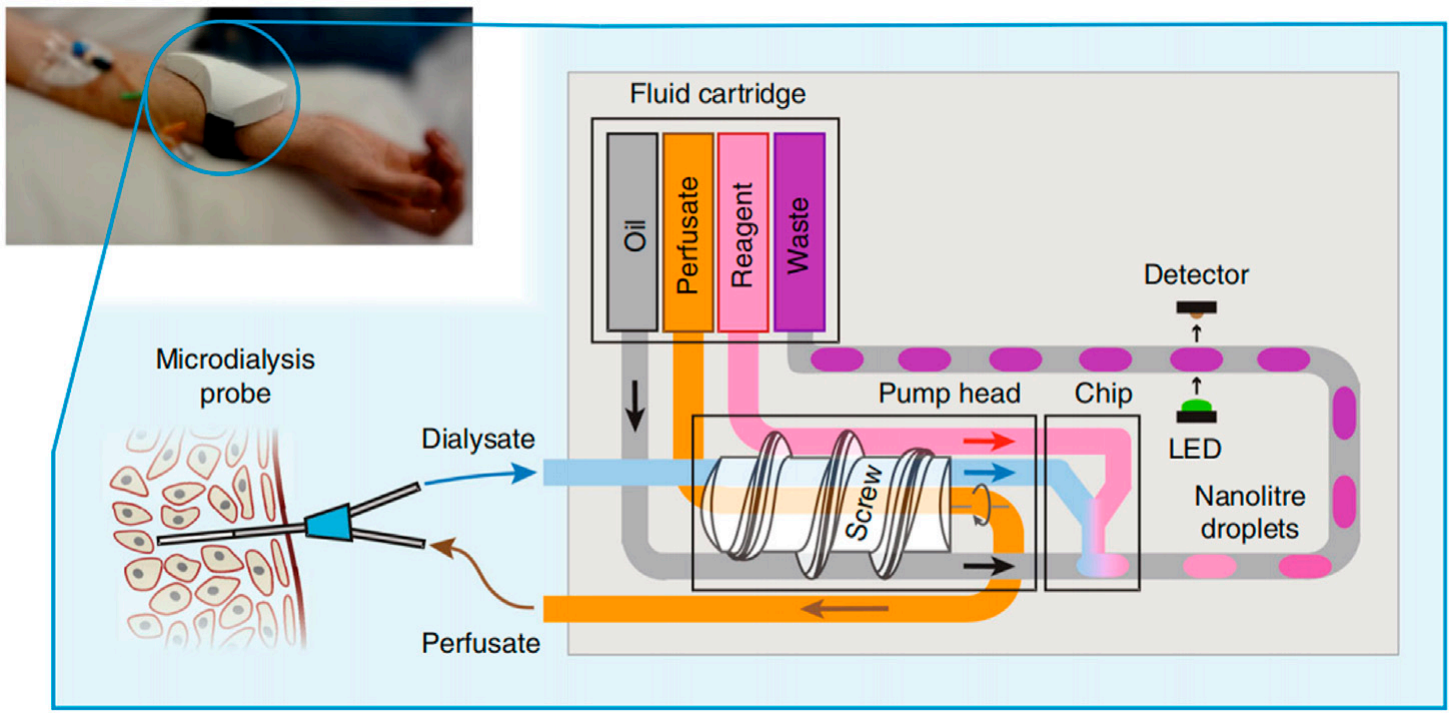
Schematic illustration of the operation of the point-of-care device (Nightingale et al., 2019).

Nightingale et al. proposed a fully integrated wearable microfluidic sensor (Nightingale et al., 2019). This sensor could provide accurate, high-resolution real-time continuous measurement in a small wearable software package, and researchers could monitor the glucose and lactate levels in healthy volunteers in real time by the sensor. The sensor could not only use droplets as *in situ* chemical analysis of the microreactor but also provide accurate, precise, and robust flow sampling and control. In the future, when it is used in combination with physical sensors, physical characteristics and biochemical data can be obtained at the same time. This rich, high-quality, and multimodal data will help in the development of accurate and personalized medical care (Figure 8).

However, as the interstitial fluid is hard to access as compared with other biofluids and the collection must be invasive, it needs further research to be applied practically. The application of microneedles is a promising method to minimize the needle wound. The poor adhesion and hydrophilicity of traditional porous polymer microneedle hinder it from further application. In Liu et al., a mild and simple poly(ethylene glycol) (PEG) and polydopamine (PDA) coating method was developed to fabricate polymer microneedles for dermal ISF extraction (Liu et al., 2021b). Owing to the anti-adhesion and hydrophilicity of PEG, the microneedle exhibited little target molecular adhesion, high fluid extraction speed, and excellent hydrophilicity. This research paved the way for microneedle-based off-line analysis in POCT and has demonstrated that the glucose concentration in the interstitial fluid extracted by the porous PDA@PEG-coated microneedles and the value determined with a glucometer in venous blood had no discernible difference.

### Urine

Glucose concentration in urine is also a significant indicator of diabetes. Because urine glucose monitoring is non-invasive and for elder patients with diabetes, glycosuria may occur with the complications of kidney disease, monitoring glucose levels in urine also attracts reasonable attention (Chen et al., 2019; Ghosh et al., 2020).

**FIGURE 9:**
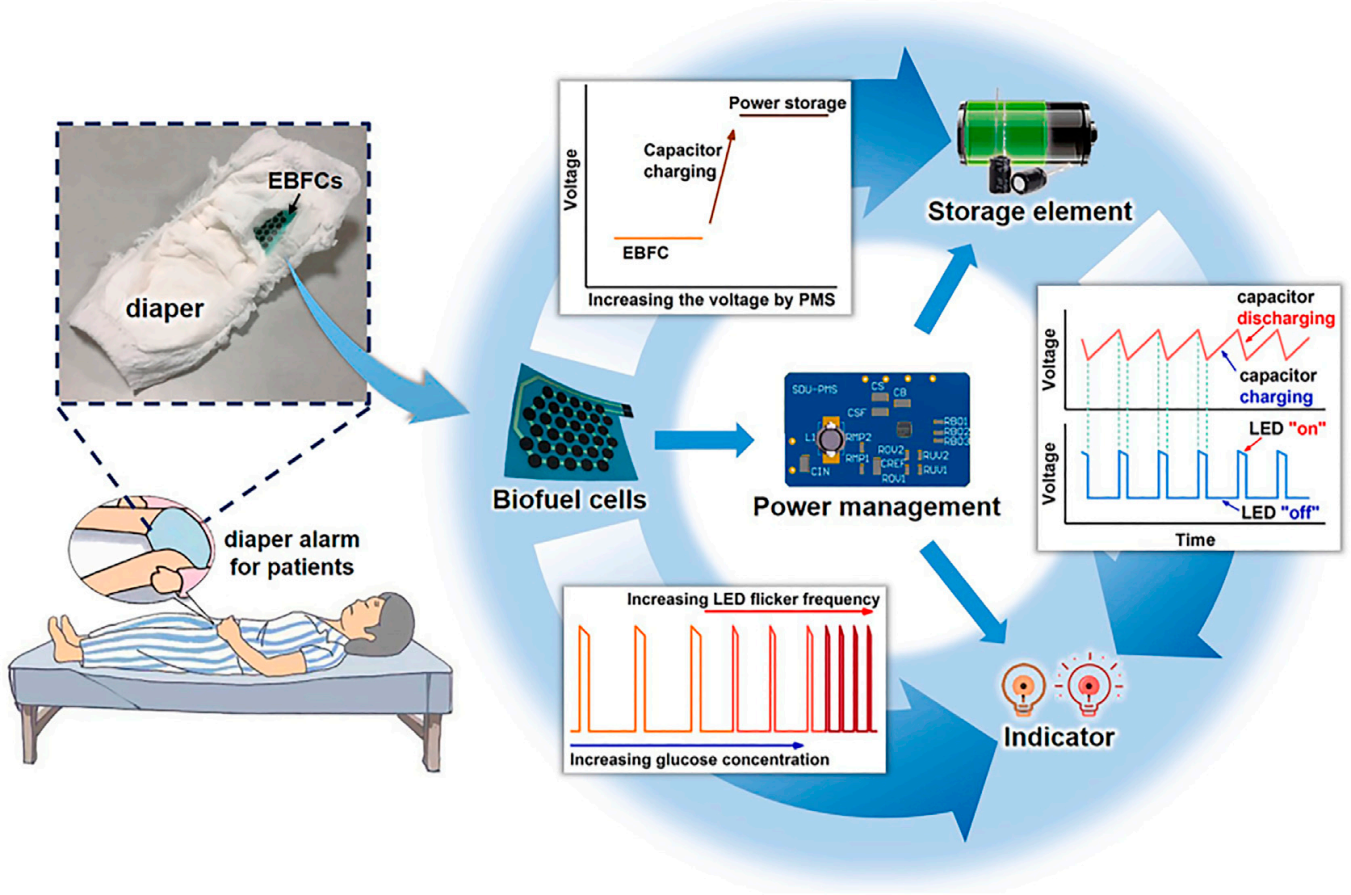
Schematic diagram illustrating the circuit illustration of the alarm glucose monitoring system and components of the wearable urine glucose biosensor system (Zhang et al., 2021a).

In Zhang et al., a wearable biosensor with the ability to detect glucose in urine was integrated with the diaper (Zhang et al., 2021a). An enzymatic biofuel cell (EBFC) with the ability to generate electricity was also integrated with the sensor to power the whole system. Additionally, a power management system (PMS) was connected with an EBFC with a power density of 220 μWcm^−2^ to store the power generated and a light-emitting diode to indicate the concentration of glucose in urine. As a result, this biosensor system displayed satisfactory anti-interference capability and provided a novel way for the urine glucose sensor to be applied for wearable point-of-care health-care devices (Figure 9).

However, urine cannot be obtained continuously, so it is hard to achieve the continuous characteristic for a wearable point-of-care urine glucose sensor. As a result, there are fewer research studies addressing this, and further studies are needed.

## Conclusion

Wearable sensors toward point-of-care monitoring of glucose in biofluids attract great attention of researchers as point-of-care testing is generally easy to use, portable, inexpensive, and non-invasive and thus causes less discomfort for users and patients. In this review, they are discussed according to six detection targets, such as blood, sweat, saliva, tears, interstitial fluid, and urine, and are tabulated in Table 1. The detection of glucose in blood is the most promising method to diagnose and monitor diabetes. Blood is the only recognized biofluid that is applied to detect glucose in daily life and is regarded as the “gold standard” for glucose measurement. Therefore, the technique is well developed, and there exist a large number of studies on wearable blood glucose sensors. Most of them are still invasive, thus leading to discomfort for users as well as adding the risk of being infected. There exists a work reported by Joshi et al. onthe non-invasive wearable point-of-care sensor for the detection of glucose in blood. However, as the sensor is based on near-infrared (NIR) spectroscopy, accuracy will be not as good as invasive glucose detection in blood and glucose detection in other biofluids, for example, sweat, saliva, tears, and interstitial fluid. Research studies toward sweat are the most among all six biofluids as sweat is easy to access and will cause no discomfort. Besides, research studies toward glucose detection in tears are increasing because some important advancements have been made to develop the wearable and point-of-care tear glucose sensor based on soft contact lens which causes less discomfort and is welcoming for people with myopia. The wearable point-of-care sensors toward the measurement of glucose in saliva need further studies and cannot be applied widely as the collection of saliva is in the mouth and will cause discomfort. Furthermore, in some cases, saliva needs pretreatment of filtration or dilution. Besides comfort and convenience, saliva is a challenging biofluid for electrochemical measurements as the concentration of biomarkers is always much low in saliva and the composition of saliva is variable in distinct cases. The research of the wearable point-of-care sensor toward the measurement of the glucose in interstitial fluid and urine is in the initial stage and needs more studies because interstitial fluid is hard to obtain and urine cannot be obtained continuously.

**TABLE 1:** Summary of wearable glucose sensors in point-of-care testing.

Biofluid	Wearable glucose sensor	Sensing method	Advantages	Refs
Blood	Wearable non-enzymatic glucose sensor	Non-enzymatic electrocatalytic reaction	• High selectivity	Hekmat et al. (2021)
			• Acceptable repeatability	
			• Long-term stability	
	Non-invasive continuous serum glucose device	Short near-infrared (NIR) spectroscopy	• Non-invasive	Joshi et al. (2020)
			• Precise	
			• Cost-effective	
Sweat	Flexible spliced self-powered sensor	Colorimetric measurements	• Self-powered	Zhang et al. (2018a)
			• Facile	
			• No need for other instruments	
	Cloth-based electrochemical sensor	Enzymatic electrocatalytic reaction	• Prominent stability	Zheng et al. (2021)
			• Reproducibility	
			• Selectivity	
			• Continuous monitoring	
	Cotton thread/paper-based microfluidic sensor	Colorimetric measurements	• Single use	Xiao et al. (2019b)
			• Excellent compatibility	
	AI/ML-enabled 2-D-RuS_2_ nanomaterial–based multifunctional sensor	Impedance change measurements	• High speed and accuracy	Veeralingam et al. (2020)
			• Prominent reusability and stability	
			• Continuous monitoring	
			• Excellent calibration	
			• Wide sensing range and low detection limit	
	Patch-based strip-type disposable sensor	Enzymatic electrocatalytic reaction	• Effective	Lee et al. (2017)
			• Closed-loop	
			• Streamlined structure	
	Nanostructured rGO-based sensor	Enzymatic electrocatalytic reaction	• Large detection range	Xuan et al. (2018)
			• Fast response	
			• High sensitivity and linearity	
Saliva	Microfluidic paper-based sensor	Colorimetric measurements	• No pretreatment steps	Tian et al. (2016)
			• Easy to produce	
			• Partially recyclable	
	Co-MOF/CC/paper hybrid button-sensor	Non-enzymatic electrocatalytic reaction	• Easy to produce	Wei et al. (2021)
			• High environment tolerance	
			• Good sensitivity	
Tears	Glucose sensor based on gelated colloidal crystal	Colorimetric measurements	• Superior portability and biocompatibility	Ruan et al. (2017)
	Glucose sensor based on MoS_2_ nanosheet	Enzymatic electrocatalytic reaction	• Facile fabrication process	Guo et al. (2021)
			• Mechanical stability	
			• Remarkable biocompatibility	
	Smart contact lenses for both continuous glucose monitoring	Enzymatic electrocatalytic reaction	• Remarkable biocompatibility	Keum et al. (2020)
ISF	Fully integrated wearable microfluidic sensor	Colorimetric measurements	• High resolution	Nightingale et al. (2019)
			• High accuracy	
			• Real-time monitoring	
Urine	Integrated with EBFC, PMS, and an LED	Enzymatic electrocatalytic reaction	Self-powered	Zhang et al. (2021a)

Note: AI/ML: artificial intelligence/machine learning; Rgo: reduced graphene oxide; co-MOF/CC/paper: cobalt metal–organic framework modified carbon cloth; ISF: interstitial fluid; EBFC: enzymatic biofuel cell; PMS: power management system; LED: light-emitting diode.

In the future, with the development in the material, power supply, and data transmission area, the wearable point-of-care glucose sensors will be more miniaturized, accurate, and self-powered. With the help of these wearable point-of-care glucose sensors, the traditional blood glucose test used most widely nowadays will be replaced, and because of the non-invasive characteristic of the novel test, patients will have less reluctance toward the glucose test. Besides the comfort, long-term monitoring of glucose can be achieved, and the obtained data will be transmitted to clinical institutions as soon as possible so that patients with diabetes can get alert and obtain professional advice from clinical personals on time. Moreover, users can have the right of choice toward the kinds of detection biofluid in the future. For example, users with myopia can choose a sensor based on contact lenses, while users with tooth disease can use a saliva-based glucose sensor.
